# Diffuse Large B-cell Lymphoma at the Base of the Tongue: A Rare Diagnostic Entity in Imaging

**DOI:** 10.7759/cureus.107749

**Published:** 2026-04-26

**Authors:** Soufiane Bigi, Said Adnor, Zakaria Chahbi, Hajar El Agouri, Soukaina Wakrim

**Affiliations:** 1 Radiology, Souss Massa University Hospital, Agadir, MAR; 2 Radiology, Hospices Civils de Lyon, Lyon, FRA; 3 Radiology, Souss Massa University Hospital, Ibn Zohr University, Agadir, MAR; 4 Pathology, Military Hospital Oued Eddahab, Faculty of Medicine and Pharmacy, Ibn Zohr University, Agadir, MAR

**Keywords:** fdg pet, head and neck malignancy, magnetic resonance imaging, non-hodgkin lymphoma, oropharyngeal lymphoma, r-chop chemotherapy, tongue base

## Abstract

Primary lymphoma of the tongue base is exceptionally rare and often presents with nonspecific clinical features, making early diagnosis challenging. We report the case of a 47-year-old male, a chronic smoker with poor oral hygiene and no notable medical history, who presented with a painless mass at the base of the tongue and mild lingual discomfort. Initial empirical antibiotic therapy for a presumed abscess was ineffective. Magnetic resonance imaging revealed an infiltrative lesion with ipsilateral submandibular lymphadenopathy. Histopathology confirmed diffuse large B-cell lymphoma (DLBCL). Fluorodeoxyglucose (FDG)-PET/CT staging demonstrated localized disease (Ann Arbor stage IEA). The patient was treated with six cycles of rituximab, cyclophosphamide, doxorubicin (hydroxydaunorubicin), vincristine (Oncovin), and prednisone (R-CHOP) immunochemotherapy and intrathecal methotrexate prophylaxis. Post-treatment imaging confirmed complete remission. Primary involvement of the tongue base by DLBCL is an uncommon presentation that can mimic benign or infectious processes. Imaging and histopathology play key roles in diagnosis, while systemic immunochemotherapy remains the mainstay of treatment. Early consideration of lymphoma in tongue base masses unresponsive to medical therapy enables timely diagnosis and effective management.

## Introduction

Non-Hodgkin lymphomas (NHL) represent a heterogeneous group of malignant neoplasms derived from B, T, or natural killer (NK) lymphocytes. Diffuse large B-cell lymphoma (DLBCL) is the most frequent and aggressive subtype, accounting for 30-40% of adult NHL cases [1,2]. While most cases arise in lymph nodes, 20-30% present in extranodal locations, with the head and neck region ranking second after the gastrointestinal tract [3,4].

Primary involvement of the tongue base is extremely rare, usually presenting with nonspecific symptoms, such as dysphagia, dysphonia, or a mass sensation, which may mimic benign or infectious processes and delay diagnosis [1-5]. Imaging plays a pivotal role in assessing tumor extension, though definitive confirmation relies on histopathology and immunohistochemistry.

We report a rare case of primary DLBCL of the tongue base and emphasize the diagnostic and therapeutic contributions of imaging in this unusual localization.

## Case presentation

A 47-year-old male, a chronic smoker for over 20 years with poor oral hygiene and no notable past medical history, presented with a painless swelling at the base of the tongue associated with mild lingual discomfort. Initial clinical evaluation suggested a subcentimetric abscess, and the patient was treated empirically with broad-spectrum antibiotics and antiseptic mouth rinses. No clinical improvement was observed, and the lesion progressively increased in size.

**Figure 1:**
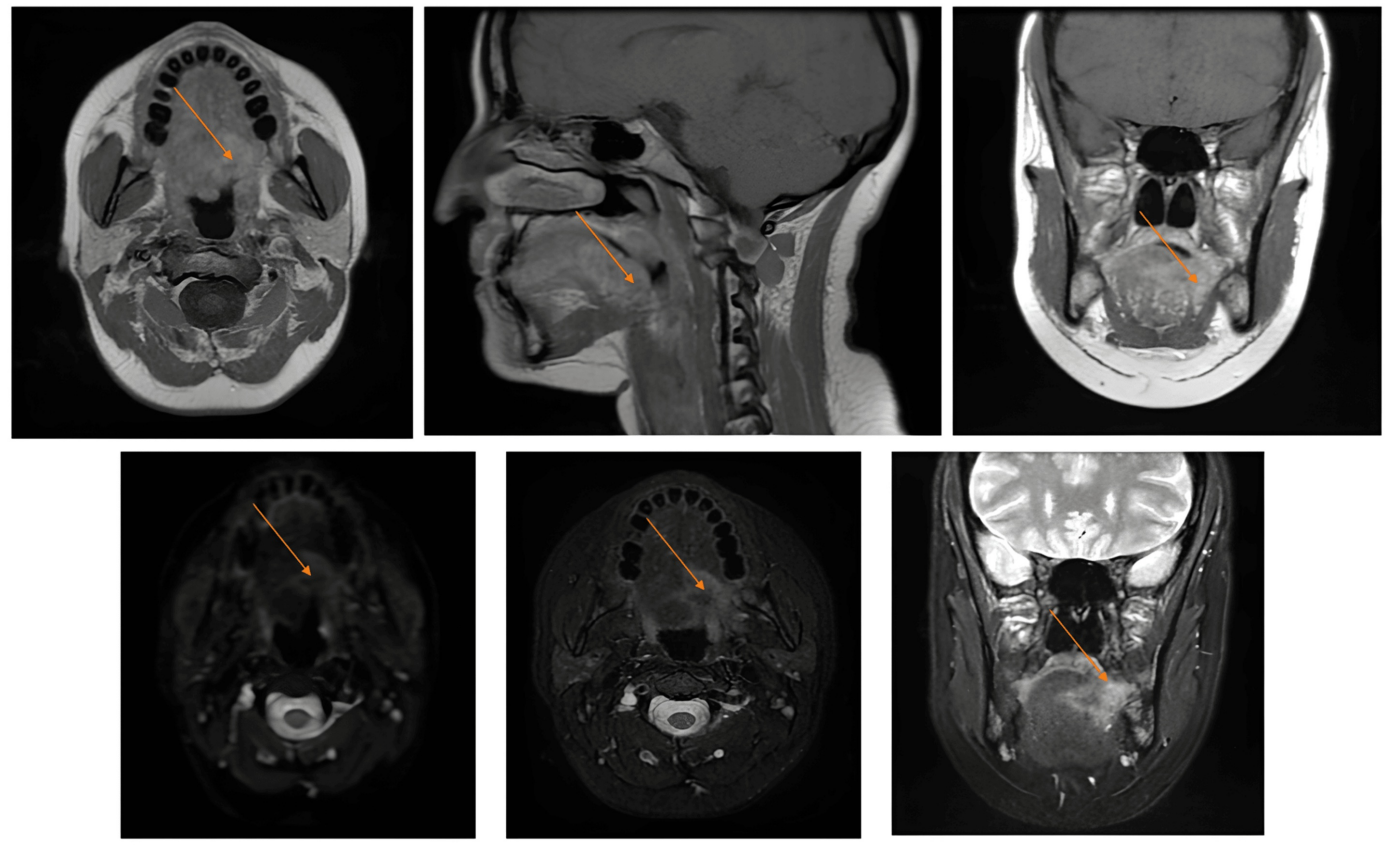
MRI images showing the process at the base of the tongue

On re-evaluation, a firm, fixed nodular lesion was detected at the tongue base. It was tender on palpation but showed no mucosal ulceration. MRI of the neck demonstrated an infiltrative mass at the tongue base with ipsilateral submandibular lymphadenopathy (levels IA and IB) (Figure 1).

**Figure 2:**
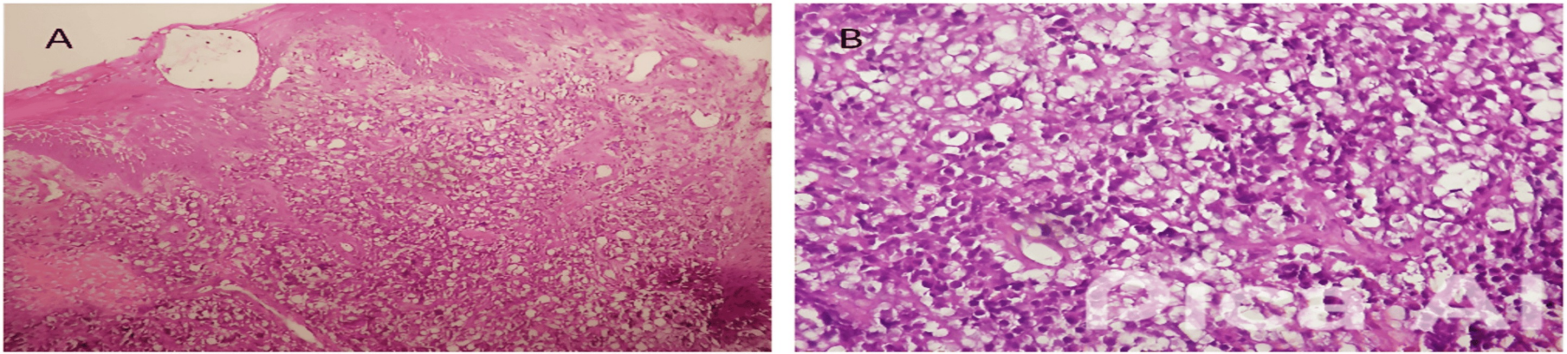
Photomicrographs showing the oral mucosa biopsy with dense lymphoid proliferation composed of large cells arranged in a diffuse pattern of moderate eosinophilic cytoplasm, with marked nuclear pleomorphism and high mitotic activity Hematoxylin and eosin stain; original magnification ×50: Image A, x200: Image B

**Figure 3:**
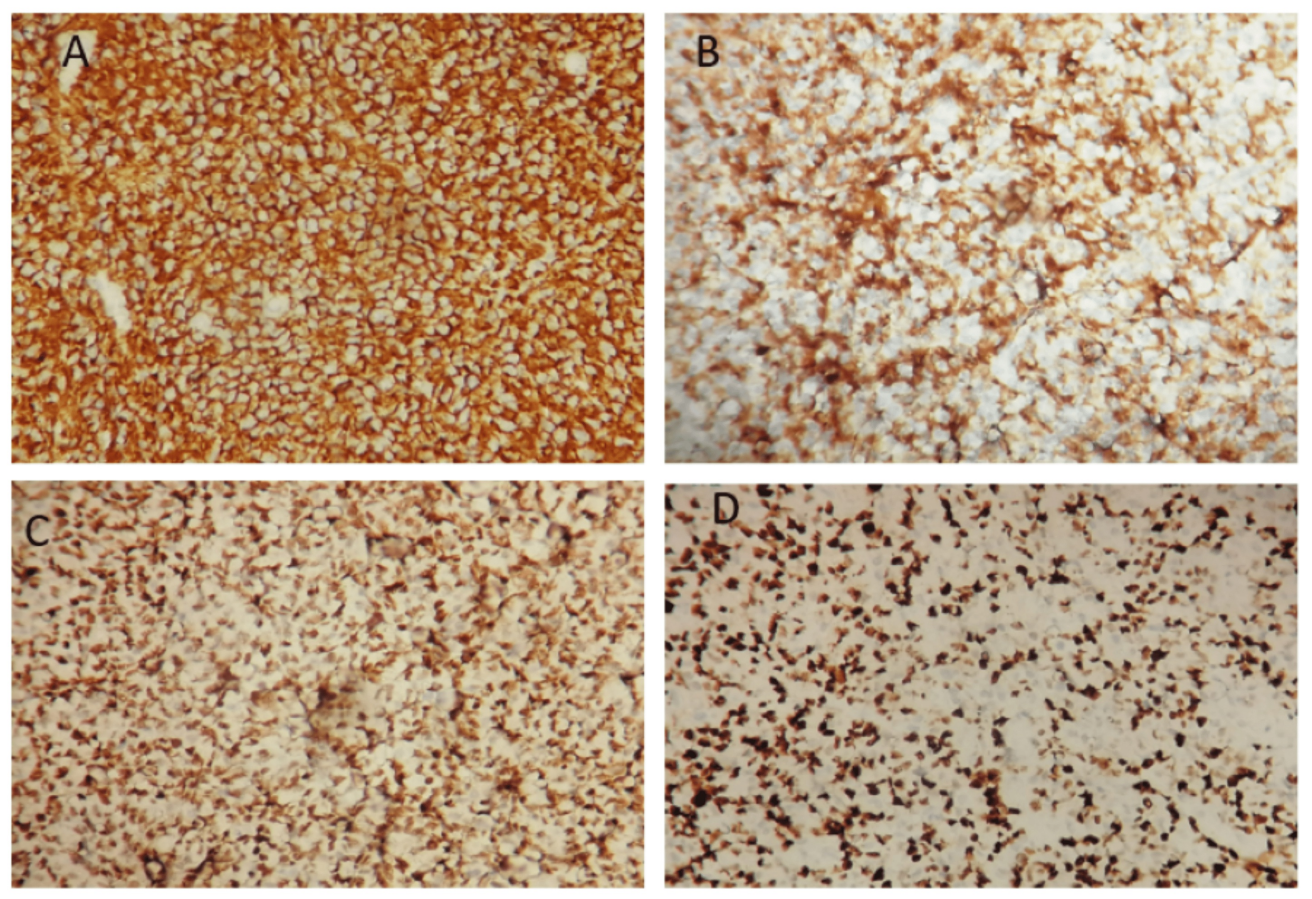
Photomicrographs showing tumor cells with diffuse immunostain to CD20 Image A: Immunochemistry stain, original magnification ×200), CD10 (Image B: Immunochemistry stain, original magnification ×200), Bcl6 (Image C: Immunochemistry stain, original magnification ×200), and high Ki67 proliferative index (Image D: Immunochemistry stain, original magnification ×200)

**Figure 4:**
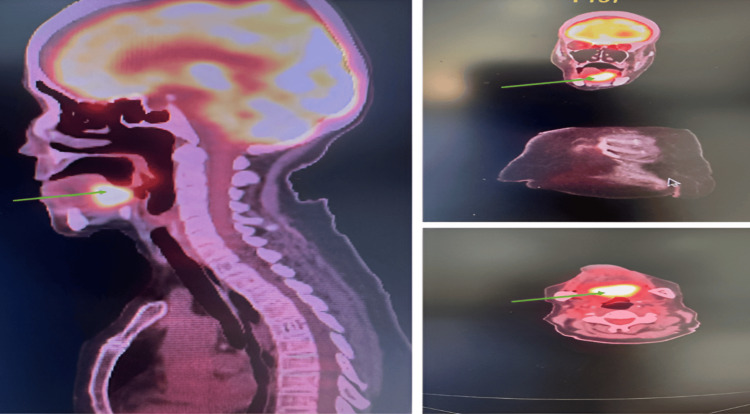
PET/CT images showing the hyperfixation process at the base of the tongue

A biopsy was performed without complications. Histopathology and immunohistochemistry confirmed DLBCL (Figures 2-3). Staging Fluorodeoxyglucose (FDG)-PET/CT revealed intense hypermetabolic activity at the primary tumor, with no evidence of distant disease (Figure 4). Laboratory results showed mildly elevated lactate dehydrogenase (LDH), with normal hematological, renal, and hepatic profiles (Table 1). A bone marrow biopsy was negative. The disease was classified as stage IEA in the Ann Arbor staging system [6].

**Table 1:** Laboratory findings at the time of diagnosis ALT: alanine transaminase; AST: aspartate aminotransferase; LDH: lactate dehydrogenase

Parameter	Result	Reference Range	Interpretation
Hemoglobin	13.5 g/dL	13-17 g/dL	Normal
White blood cells	6.8 × 10⁹/L	4.0-10.0 × 10⁹/L	Normal
Platelets	245 × 10⁹/L	150-400 × 10⁹/L	Normal
LDH	280 U/L	140-250 U/L	Mildly elevated
Creatinine	0.9 mg/dL	0.6-1.3 mg/dL	Normal
ALT	28 U/L	<40 U/L	Normal
AST	25 U/L	<40 U/L	Normal
Total bilirubin	0.8 mg/dL	0.3-1.2 mg/dL	Normal

The patient was treated with six cycles of rituximab, cyclophosphamide, doxorubicin (hydroxydaunorubicin), vincristine (Oncovin), and prednisone (R-CHOP), administered every three weeks. Given the localization within a high-risk neuro-meningeal zone, intrathecal methotrexate prophylaxis was added. The treatment was well tolerated, with no major infectious or hematological complications. Post-treatment MRI and FDG-PET/CT confirmed complete remission, both morphologically and metabolically. Clinically, the lingual swelling resolved, and symptoms disappeared.

## Discussion

DLBCL is the most common and aggressive NHL subtype, representing 30-40% of adult cases [2]. Although nodal involvement predominates, 20-30% of cases present in extranodal sites, with the oropharyngeal region being the second most frequent after the gastrointestinal tract [3,5].

Primary involvement of the tongue base is exceedingly rare and poses diagnostic challenges. Initial clinical manifestations are often nonspecific and may mimic benign or infectious conditions, as in our case, where the lesion was initially mistaken for an abscess [1,4]. Clinical examination alone is insufficient to differentiate DLBCL from squamous cell carcinoma or other solid tumors.

Imaging plays an essential role in disease assessment. MRI provides excellent evaluation of locoregional extension and anatomical relationships. FDG-PET/CT is indispensable for staging and monitoring treatment response, with high sensitivity and specificity in DLBCL [7,8].

The gold standard for diagnosis remains histopathology with immunohistochemistry, typically demonstrating B-cell markers (CD20, CD79a) and a high proliferation index (Ki-67) [9].

Standard treatment consists of immunochemotherapy with R-CHOP, which has significantly improved prognosis, even in extranodal forms [10]. For high-risk head and neck sites, such as the tongue base, prophylactic intrathecal methotrexate may be indicated to prevent CNS dissemination [11].

Localized DLBCL of the oropharyngeal region generally carries a favorable prognosis when diagnosed early and treated appropriately. Our patient achieved complete remission after six cycles of R-CHOP, illustrating the effectiveness of this regimen.

## Conclusions

Primary DLBCL of the tongue base is an exceptionally rare entity, often associated with diagnostic delays due to its misleading clinical presentation. This case underscores the importance of considering lymphoma in the differential diagnosis of lingual masses, particularly those resistant to empirical therapy.

Imaging, combined with histopathology, is crucial for accurate diagnosis and treatment planning. Early recognition and appropriate multidisciplinary management are key to achieving favorable outcomes.
